# Current Perspectives regarding Stem Cell-Based Therapy for Alzheimer's Disease

**DOI:** 10.1155/2018/6392986

**Published:** 2018-03-01

**Authors:** Kyeong-Ah Kwak, Seung-Pyo Lee, Jin-Young Yang, Young-Seok Park

**Affiliations:** ^1^Department of Oral Anatomy, Dental Research Institute, School of Dentistry, Seoul National University, Seoul, Republic of Korea; ^2^Department of Dental Hygiene, Daejeon Institute of Science and Technology, Daejeon, Republic of Korea

## Abstract

Alzheimer's disease (AD), a progressive neurodegenerative disorder featuring memory loss and cognitive impairment, is caused by synaptic failure and the excessive accumulation of misfolded proteins. Many unsuccessful attempts have been made to develop new small molecules or antibodies to intervene in the disease's pathogenesis. Stem cell-based therapies cast a new hope for AD treatment as a replacement or regeneration strategy. The results from recent preclinical studies regarding stem cell-based therapies are promising. Human clinical trials are now underway. However, a number of questions remain to be answered prior to safe and effective clinical translation. This review explores the pathophysiology of AD and summarizes the relevant stem cell research according to cell type. We also briefly summarize related clinical trials. Finally, future perspectives are discussed with regard to their clinical applications.

## 1. Introduction

According to reports from the Alzheimer's Association, there are approximately 50 million people with dementia worldwide, accounting for approximately 800 billion dollars. Alzheimer's disease (AD) is the most frequent form of dementia, which shows clinical manifestations of progressive loss of memory and impairment of cognitive functions. The disease was first reported by Alois Alzheimer in 1907 [1]. As life expectancy rises and the population ages, the social burden of AD is predicted to soar [2, 3]. Alzheimer's disease is multifactorial; therefore, it is difficult to determine its exact pathophysiologic mechanism [4]. However, synaptic failure is the main feature that is caused by neuronal loss in the region of the brain cortex and hippocampus due to the excessive accumulation of neurofibrillary tangles and *β* amyloid (A*β*) plaques [5–8]. However, approximately one-third of patients with a documented diagnosis of AD have no radiographic signs of amyloid on PET scans [9]. Therefore, more sophisticated approaches, including imaging and pathology, are likely needed in the diagnosis of AD [10, 11].

There are two main types of AD: familial and sporadic. Familial AD comprises <5% of cases and is associated with a distinct autosomal genetic mutation associated with the amyloid precursor protein (APP), presenilin-1 (PSEN-1), and presenilin-2 (PSEN-2) [5, 12–18]. In contrast, sporadic AD accounts for the majority of cases. It typically has a late onset and is thought to result from interactions between a complex genetic profile (including apolipoprotein [ApoE4]) and environmental factors such as cardiovascular disease, depression, and lower levels of education [19, 20].

The cardinal pathologic features of AD include the accumulation of two types of misfolded proteins [21, 22]. One such protein is tau, which is a microtubule-associated protein that is important for axonal transport and structural support. When the tau protein becomes hyperphosphorylated, the microtubules lose their support and neurofibrillary tangles aggregate [23]. Although this process is closely associated with cognitive decline, tau mutations lead to frontotemporal dementia rather than AD [4]. The other important protein in AD is A*β* protein, which is the abnormal sequential cleavage product of APP. The A*β* aggregates to form senile plaques, which are known to cause calcium influx and neuronal cell death [24]. A*β* oligomers are considered to be especially detrimental to synaptic and neuronal functions and result in cognitive dysfunction [25, 26]. Mutations regarding APP and its processing are prominent characteristics of early-onset familial AD. Therefore, most patients with AD do not actually have these mutations. Instead, 60–75% of sporadic AD populations are ApoE4 carriers [27]. Several evidences support that ApoE4 has important roles in pathogenesis not only A*β* dependently but also independently [4, 28, 29]. Therefore, ApoE4 is thought to be an important gene in the semidominant inheritance of sporadic late-onset AD [14, 15].

In addition to these two specific proteins, microglial activation and subsequent inflammatory responses are thought to contribute to the neurodegenerative symptoms of AD [30, 31]. Activated microglia produce several proinflammatory cytokines, including interleukin- (IL-) 1*β* and tumor necrosis factor- (TNF-) *α*, as well as nitric oxide (NO) [32–35]. Oxidative stress and mitochondrial dysfunction have also been suggested to play a role in AD pathogenesis [36–41]. The dysfunction of the GABAergic neuronal system is thought to contribute to learning and memory deficits in patients with AD [4, 17, 42–52].

Until now, the main therapeutic strategy in AD of most drug developments has focused on facilitating amyloid clearance or preventing amyloid production [4, 53–55]. Before the amyloid pathway was proposed [56], clinical trials using cholinesterase inhibitors were performed based on the notion that memory is closely related to cholinergic systems [57–59]. Therefore, a number of small molecules and antibodies targeting the amyloid cascade have been developed and investigated in clinical trials [53, 54, 60–67]. Unfortunately, the results from almost all of these trials were far from satisfactory. There is little evidence to support the efficacy of such treatments [68]. Dementia prevention trials have also used many other agents, including antihypertensive drugs, NSAIDs, vitamin E, selenium, and *Ginkgo biloba* [69–73], all of which had no effect on reducing the risk of AD.

The conventional mediations investigated have yielded no clinical benefits for AD. Therefore, there is a large unmet need for patients suffering from AD. Recently, stem cells have gained interest as a potential alternative to conventional medicines or surgery. Several attempts have been made to appreciate the clinical applications of stem cells with regard to an advanced understanding of the cellular and molecular mechanisms of neuroregeneration and neurodegeneration [74–78]. Stem cell-based therapy is a potentially promising strategy in the treatment of various neurologic disorders that do not otherwise have any effective treatments, including stroke, Parkinson's disease, Huntington's disease, amyotrophic lateral sclerosis, and AD [79–83]. This article reviews the current literature according to stem cell type and discusses the future of stem cell-based therapy in Alzheimer's disease.

## 2. Expected Mode of Action

Stem cells can incorporate into existing neural networks [84]. They also secrete a variety of neurotrophic factors to modulate neuroplasticity and neurogenesis [77, 78], which appear to increase brain acetylcholine levels, ultimately leading to improved memory and cognitive function in an animal model [75]. The primary modes of actions of stem cell-based therapy can be categorized into endogenous and exogenous ways depending on the mechanisms of action [19]. Traditionally, cell-based therapies have sought to replace damaged tissue through tissue repopulation either by transdifferentiation or by direct participation of infused stem cells [84]. However, the current understanding suggests that engrafted stem cells are not a main source for newly generated neurons [76, 85–90]. Furthermore, unlike in Parkinson's disease, AD is characterized by the death of various distinct nerve cell types. This variability precludes the feasibility of transplantation of specific mature cell types.

Rather than using the cell replacement paradigm, therefore, there is a growing interest in the stimulation of endogenous repair using paracrine effects. The trophic support provided by transplanted stem cells improves the microenvironment and promotes the survival of affected/remaining nerve cells [3, 91]. Using this strategy, the primary target to stimulate hippocampal neoneurogenesis (in order to compensate for neurodegeneration) is the upregulation of resident neural stem cell niches. Hippocampal neoneurogenesis is believed to play a key role in memory and learning. Neurotrophic factor (BDNF), nerve growth factor (NGF), insulin growth factor-1 (IGF-1), and vascular endothelial growth factor (VEGF) are suggested paracrine mediators from transplanted stem cells [92]. Unfortunately, the potential for neurogenesis in humans decreases substantially with older age, which is primarily when AD occurs [93, 94]. In addition, the modulation of inflammation has been proposed as another mechanism of action [76].

## 3. Stem Cell Types

### 3.1. Embryonic Stem Cells

Human embryonic stem cells (ESCs) were first characterized in 1998 from the inner mass of the blastocyst [95]. If their pluripotency could be accurately controlled into the desired neural phenotypes, no other alternative cells would replace them as a better cell source for cell replacement strategies. In vitro attempts to differentiate ESCs into several specific neural cell types have been successful, including dopaminergic neurons [96–102]. Indeed, an ex vivo slice culture study reported stable generations and the functional integration of cholinergic neuron from human ESCs [103]. Despite the ongoing preclinical studies, there are a number of issues that remain with the current technologies, including tumor formation, phenotype instability, and low survival rate of transplanted cells [104, 105]. Furthermore, there are ethical and immunogenic limitations that may preclude the clinical usage of ESCs. In fact, given the ethical policies and regulation, there are few clinical trials that have involved ESCs [106, 107].

### 3.2. Induced Pluripotent Stem Cells

Induced pluripotent stem cells (iPSCs) were first developed from mouse fibroblasts in 2006. These cells are reprogrammed into a state of pluripotency that is similar to that of ESCs [108]. iPSCs are thought to be able to differentiate into a variety of cells, including neurons [109] and neurospheres [110]. Several encouraging reports have been published showing that some neuronal subtypes can be generated and automated using iPSCs [84, 111–114]. For example, iPSC-derived glia could be used for research regarding inflammatory response in AD [115]. Another study used iPSCs to derive macrophages that could express neprilysin, the A*β*-degrading protease [116].

Despite this promising evidence, however, the following unresolved issues regarding iPSC usage constitute big hurdles to its clinical application: teratoma formation, long-term safety and efficacy, tumorigenicity, immunogenicity, patient-derived genetic defects, and optimal reprogramming [117–121]. Ethical guidelines and standards have been developed regarding the use of iPSCs [122, 123]. Therefore, applications of iPSCs in AD, until now, have been more focused on the development of cell-based disease models than on treatments [124–130]. Basal forebrain cholinergic neurons have been of special interest, as they demonstrate dysfunction in early AD [131]. In later stages of AD, strategies using iPSCs should be more elaborated due to widespread degeneration [6, 132]. Unfortunately, prior studies have found that human iPSC lines have only a 10–50% differentiation potential for neurons, as compared to ESCs, which have a nearly 90% differentiation potential [133, 134].

### 3.3. Neural Stem Cells

In the adult brain, multipotent neural stem cells (NSCs) reside in the subgranular zone (SGZ) and subventricular zone (SVZ) [81]. They can differentiate into a variety of cell types, including neurons, astrocytes, and oligodendrocytes. NSCs can also be derived from fetal and postmortem neonatal brain tissues [76] or differentiated from ESCs and iPSCs [135–137]. In animal AD models, transplanted NSCs differentiated into mature brain cell types [138–141]. For successful neuronal replacement, the grafted cells should be distributed throughout the affected tissue (maintaining its original identity) and then integrated into the host brain's functional environment [142]. However, it is unknown if NSCs can generate into specific neural cell types. Interestingly, the migration and differentiation of grafted NSCs appeared substantially influenced by the recipient environment [143, 144]. However, there is frequent unwanted differentiation into nonneuronal glial cell types reported with NSCs [140].

It is not clear how much neuronal replacement contributes to the beneficial effects of NSC transplantation [140, 145, 146]. As is the case in the transplantation of other stem cells, the paracrine effect after NSC transplantation has gained more support than has cell replacement [75, 147]. In particular, brain-derived neurotrophic factor (BDNF) secreted from NSCs is essential for rescuing cognitive function in AD [148, 149]. In addition, NSC transplantation has been reported to have neuroprotective, neuroregenerative, and/or immune modulatory roles [148, 150–153]. Tumorigenesis and functional recovery warrant further investigations using NSC transplantation [145].

The NSCs can be induced from other cells. The generation of induced NSCs (iNSCs) from fibroblasts, astrocytes, and Sertoli cells has been reported [154–161]. NSCs that are derived from ESCs have also been investigated and differentiated into astrocyte-like cells [162]. However, the in vivo viability of iNSCs after transplantation is still considered unpredictable [154, 156–158, 163]. As a modified strategy, therefore, NSCs can be used as delivery vehicles to carry therapeutic agents such as neprilysin [164, 165]. Using NSC-based therapy for drug delivery, rather than for neuronal replacement, has gained interest recently [74, 76, 135, 149, 166].

### 3.4. Mesenchymal Stem Cells

Mesenchymal stem cells (MSCs) have received special interest in the treatment of AD given their excellent accessibility, relative ease of handling, extensively studied characteristics, and broad range of differentiating potential (including neuronal cells) [78, 167]. MSCs are additionally advantageous as cell-based therapies given that they can be administered intravenously, exhibit blood-brain barrier penetration, have low tumorigenicity, and elicit less of an immune response (than do other cell-based therapies) [168, 169].

Unfortunately, there is little evidence for the functionality of MSC-derived neurons in vivo with low rates of neuronal differentiation [170]. Rather than for neuronal replacement, the beneficial effects of MSCs seemed to be mediated by their secreted factors, which stimulate the proliferation, differentiation, and survival of the neurogenic niche [168, 171–178]. Well-known anti-inflammatory and immune modulatory characteristics are also presumed to contribute to recovery, which involves a number of cytokines [171, 175, 177, 178]. Notably, the homogeneity of MSCs is suspected in their phenotypic expression and differentiation [179].

There is a wide variety of sources from which MSCs are acquired. Bone marrow-derived MSCs (BM-MSCs) have been most widely investigated since a long time ago [162]. BM-MSCs gain their immunomodulatory ability through the release of soluble factors, including IL-6, IL-10, TGF-*β*, and PGE2 [180–182]. They are known to inhibit the functioning of monocyte-derived dendritic cells and to alter the natural killer cell phenotype [183, 184]. Adipose tissue is one of the most advantageous sources of MSCs. Adipose tissue-derived MSCs (AT-MSCs) can differentiate into neuron-like and astrocyte-like cells [185]. They seem to share a common transcriptional profile for stemness with BM-MSCs [186, 187]. AT-MSCs also secrete many neurotrophic factors [188–193]. Finally, umbilical cord blood-derived MSCs (UCB-MSCs) can differentiate into neuron-like cells. These cells have been studied in an AD mouse model, as well as clinically [194]. One suggested mechanism of action is the activation of M2-like microglia [177, 195].

### 3.5. Other Cells

Several other stem cells have been investigated with regard to their potential in neuronal regeneration, including neural crest stem cells [196–200], hematopoietic stem cells [201], human dental pulp stem cells [202–204], and olfactory ensheathing cells [205–210]. Remarkably, the somatic cell nuclear transfer procedure involving olfactory ensheathing cells is another promising technology via the intranasal route [211–214].

## 4. Clinical Trials

There was sufficient animal model evidence for MSC-based therapies to approve the initiation of clinical trials in patients with AD since 2011 (Table 1). Intravenous infusion is the most preferred delivery method of stem cells, and UCB-MSCs were the most frequently used cell source. According to Kim et al., human UCB-MSCs were transplanted into the hippocampus and precuneus stereotactically. Although there were no severe adverse events, the group did not identify any significant clinical efficacy in cognitive decline (ClinicalTrials.gov, NCT01297218, NCT01696591) [175]. Furthermore, there were no changes in pathology or observed neuroprotective effects [175, 177, 178]. These results may be partly due to neuroimaging, which can be an insensitive modality for detecting such changes compared to that of postmortem biochemical analyses.

Three additional clinical trials using UCB-MSCs are currently underway. One involves intravenous injection in an open-label phase I/II study (NCT01547689), while another involves intravenous infusion in a double-blind randomized placebo-controlled study (NCT02672306). The third study involves intraventricular injection of the Ommaya reservoir system (NCT02054208). As an alternative source of MSCs, one trial will assess the outcome of AT-MSCs (NCT02912169). A phase 2A study (NCT02600130, NCT02833792) will utilize the intravenous administration of allogenic ischemia-tolerant allogeneic BM-MSCs grown in hypoxic conditions [215].

## 5. Discussion

Cognitive declines in AD result from the loss of neurons and neuronal processes, which result from diverse factors. The pathways of toxic protein synthesis and degradation in AD have been rigorously investigated to determine the most effective disease management [132]. To date, efforts to develop target-specific drugs have not succeeded. The progressive and devastating nature of AD requires breakthrough therapy to satisfy the unmet needs of patients. Cell-based therapies may offer a promising solution to this need. They may be able to not only reverse the progression of AD but also improve cell function.

Technologic advances have sought to generate various types of neuronal cells with glial cells. These investigations have led to the replacement and regeneration concept of stem cell-based therapies in AD. Substantial achievements have been made in animal models as a proof of concept [29, 143, 145, 149, 162, 165, 166, 216–219]. Despite the promising results of preclinical studies, human clinical trials are still in their infancy with regard to stem cell therapies. There are many more questions that must be answered prior to transferring this technology from the bench to the clinic.

There are several cardinal questions that ought to be addressed for the clinical translation of stem cell therapies, such as optimum cell source, long-term safety, and routes of delivery. The brain is an immune-privileged organ; therefore, it is important to consider immune rejection when using stem cell therapies [142]. Given that most AD patients are elderly, special caution is necessary regarding the difference in donor cell proliferation capability [216, 220, 221]. The variability of donor cells and unstandardized reprogramming methods could also pose a problem [222, 223]. The current, general concerns regarding stem cell-based therapy are as follows: tumorigenicity, immune reaction, contamination while handling, risks from genetic modification, risks of administration modality, unintended migration, unwanted transdifferentiation, infection, and death of the transplant [224–227]. There are also ethical concerns with regard to certain cell sources.

Alzheimer's disease involves the death of variable neuronal cell types. In its early phases, the hippocampal circuitry can be a major therapeutic target [19]. In advanced phases, additional neuronal subtypes become involved. Therefore, the therapeutic strategy will become more complicated as the disease progresses. In clinical trials, extremely elaborate controls must be used, unless each involved cell type is transplanted (at the same time or sequentially). In this regard, pluripotent stem cells might be advantageous in AD over another source. However, the current evidence does not suggest that cell replacement is the mechanism of stem cell-based therapy.

The iPSC approach deserves attention given its biological relevance. The major advantages of this approach are that iPSCs can be made autologous, be differentiated into intended cell types, and provide a sufficient quantity [132]. In order to advance toward successful iPSC-based therapies for AD, the following parameters must be met: establish haplobanks of HLA-typed iPSCs for off-the-shelf cell therapies [228], establish protocols to create neuronal stem cells or hippocampal neurons with appropriate surgical and tracking techniques, and establish an astrocyte-generation technique for providing trophic agents [226]. We believe that the development of individual cell tracking and real-time imaging will be essential [229–233].

Eventually, there is a need for precise manufacturing practices in the preparation and handling of transplantable cells for clinical use. Prior to their clinical use, if able to be manipulated in vitro, all grafted cells would ideally be transgenically equipped with a molecular “kill switch” that could be easily activated in the event of adverse effects. AD can be a relatively slowly progressive disease; therefore, clinical trials are expected to require many years to demonstrate success in halting or reversing disease progression. The safe and ethical future of stem cell therapies, especially for AD, will likely be slow, expensive, and tightly controlled [166]. However, given the unique nature of stem cell-based therapies, regulatory agents are needed to develop new regulatory policies to foster their appropriate development and success.

## 6. Conclusion

Alzheimer's disease is a progressive neurodegenerative disease for which there is no effective treatment currently. Stem cell-based therapies may become an effective therapeutic alternative (to conventional therapies) due to their regenerative potential. Although the mechanism of action of stem cell therapies remains incompletely elucidated, a number of preclinical studies have provided promising results. However, human clinical trials are still in their infancy. For the successful clinical translation of this technology, further relevant animal studies and clinical trials (with standardized protocols) are needed. There are many questions left unanswered regarding the safety, efficacy, ethical issues, and regulatory framework of stem cell-based therapies. There is a growing hope for patient-specific individualized stem cell-based therapy. This review attempts to provide a synopsis of stem cell-based therapy for AD in particular. We describe the pathophysiology of AD and proposed mechanisms of stem cell therapies. Preclinical results according to the cell type and clinical trials are briefly summarized. Future perspectives are also discussed.

## Figures and Tables

**Table 1:** Main clinical trials of stem cell therapy for Alzheimer's disease.

Trial number	Study phase (type)	Sponsor	The route of administration	Cell source	#	Eligibility criteria	Primary outcome measure	Secondary outcome measure	Time frame	Start date	End date	Location
NCT01297218	Phase I	Medipost Co. Ltd.	Intravenous	hUCB-MSC	9	Dementia as determined by DSM-IV criteria; probable Alzheimer's disease as determined by NINCDS-ADRDA criteria; K-MMSE score in the range of 10 to 24	Number of participants with adverse events	Changes from the baseline in ADAS-Cog at 12 weeks postdose	12 weeks	2011-02	2012-12 (completed)	Korea
NCT01547689	Phase I/II (open)	Affiliated hospital to the Academy of Military Medical Sciences	Intravenous	hUCB-MSC	30	Probable Alzheimer's disease as determined by NINCDS-ADRDA criteria; MMSE score between 3 and 20, both inclusive	Number of participants with adverse events	Changes from the baseline in ADAS-Cog at 10 weeks postdose	10 weeks	2012-03	2016-12 (active, not recruiting)	China
NCT01617577	Phase I/II (crossover)	University of South Florida	Subcutaneous	Filgrastim (G-CSF)	8	People with probable AD (by NINCDS-ADRDA criteria); MMSE score between 10 and 24	Cognitive measures including ADAS-Cog, selected CANTABS tests	None	2, 4, 14 weeks	2009-06	2012-02 (completed)	USA
NCT01696591	Phase I (case controlled)	Duk Lyul Na	Brain surgery	hUCB-MSC	14	Subjects who have enrolled in NCT01297218 and who have similar characteristics	Incidence rate of adverse events	ADAS-Cog response rate	24 months	2012-03	2013-09 (unknown)	Korea
NCT02054208	Phase I/II (randomized quadruple blind controlled)	Medipost Co. Ltd.	Intraventricular	hUCB-MSC	45	Diagnosis of probable Alzheimer type according to NINCDS-ADRDA criteria and K-MMSE score of 18–26 at visit 1	Number of subjects with adverse events	Change from the baseline in ADAS-Cog, S-IADL, K-MMSE, CGA-NPI, and so forth	24 months	2014-02	2019-07 (recruiting)	Korea
NCT02600130	Phase I	Longeveron LLC	Peripheral intravenous	Longeveron MSC	30	At the time of enrollment, be diagnosed with AD in accordance with the NINCDS-ADRDA criteria; MMSE score between 18 and 24	Incidence of any serious adverse events	Neurologic/neurocognitive assessments, ADAS-Cog 11, and so forth	2, 4, 13, 26, 39, 52 weeks	2016-08	2019-10 (recruiting)	USC
NCT02672306	Phases I, II	South China Research Center for Stem Cell and Regenerative Medicine	Intravenous	hUCB-MSC	40	A diagnosis of probable AD and mixed dementia according to the criteria of the NINCDS-ADRDA, MMSE score between 3 and 20, both inclusive	Change in ADAS-Cog score	Change in ADCS-CCGIC score, MMSE, ADCS-ADL, and so on	10 weeks	2016-05	2019-10 (not yet recruiting)	China
NCT03172117 ^∗^	Phase I/II (randomized quadruple blind controlled)	Medipost Co. Ltd.	Intraventricular	hUCB-MSC	45	Diagnosis of probable Alzheimer type according to NINCDS-ADRDA criteria and K-MMSE score of 18–26 at visit 1	Change from the baseline in ADAS-Cog	Change from the baseline in S-IADL, K-MMSE, CGA-NPI, and so forth	24 months	2017-05	2021-12 (recruiting)	Korea

# indicates the number of enrollment; AD: Alzheimer's disease; ADAS-CCGIC: Alzheimer's Disease Cooperative Study Clinician's Global Impression of Change; ADAS-Cog: Alzheimer's Disease Assessment Scale-Cognitive Subscale; CGA-NPI: Caregiver-Administered Neuropsychiatric Inventory; DSM: *Diagnostic and Statistical Manual of Mental Disorders*; hUCB-MSC: human umbilical cord blood-derived mesenchymal stem cell; K-MMSE: Korean version of Mini-Mental State Evaluation; MMSE: Mini-Mental State Evaluation; NINCDS-ADRDA: National Institute of Neurological and Communicative Disorders and Stroke and the Alzheimer's Disease and Related Disorders Association; S-IADL: Seoul-Instrumental Activities of Daily Living. ∗ is the follow-up study of clinical trial number 02054208.
